# Efficacy of a novel topical combination of esafoxolaner, eprinomectin and praziquantel against ear mite (*Otodectes cynotis*) infestations in cats

**DOI:** 10.1051/parasite/2021022

**Published:** 2021-04-02

**Authors:** Eric Tielemans, Joe Prullage, Otsuki Tomoko, Julian Liebenberg, Balázs Capári, Smaragda Sotiraki, Despoina Kostopoulou, Panagiota Ligda, Michael Ulrich, Martin Knaus

**Affiliations:** 1 Boehringer-Ingelheim Animal Health 29 Avenue Tony Garnier 69007 Lyon France; 2 Boehringer-Ingelheim Vetmedica GmbH, Kathrinenhof Research Center Walchenseestr. 8–12 83101 Rohrdorf Germany; 3 Boehringer-Ingelheim Animal Health, Missouri Research Center 6498 Jade Rd. Fulton 65251 MO USA; 4 Boehringer Ingelheim Animal Health Japan Co Ltd ThinkPark Tower, 2-1-1 Osaki Shinagawa-ku 141-6017 Tokyo Japan; 5 Clinvet International (Pty) Ltd. P.O. Box 11186 Universitas 9321 Bloemfontein Republic of South Africa; 6 Kapriol Bt. Vak Bottyán St. 1 8330 Sümeg Hungary; 7 Veterinary Research Institute, ELGO-DIMITRA Campus Thermi 57001 Thessaloniki Greece; 8 Cheri-Hill Kennel & Supply Inc. 17190 Polk Rd. Stanwood 49346 MI USA

**Keywords:** Cat, Ear mite, *Otodectes cynotis*, Esafoxolaner, Efficacy

## Abstract

Esafoxolaner, a purified enantiomer of afoxolaner with insecticidal and acaricidal properties, is combined with eprinomectin and praziquantel, nematodicidal and cestodicidal compounds, in NexGard^®^ Combo, a novel topical endectoparasiticide formulation for cats. The efficacy of this formulation was assessed against *Otodectes cynotis* in two laboratory studies conducted in South Africa and in the USA with local isolates, and in one field trial conducted in Europe. In each study, cats were randomly allocated to a placebo-treated control group and a novel formulation-treated group. In the laboratory studies, cats were treated at the minimum recommended dose; in the field trial, cats were treated at label dose. All included cats were diagnosed positive for *O. cynotis* prior to treatment by otoscopy. The main variable of efficacy was a comparison of the number of live *O. cynotis* collected in both ear canals of all cats in the treated and control groups, one month after treatment. Efficacy of the novel topical formulation exceeded 97% in the three studies. These studies demonstrated the high effectiveness of NexGard^®^ Combo in cats for the treatment of *O. cynotis* infestations. No health abnormalities were attributed to the treatment in any of the studies.

## Abstract (fr)

L’esafoxolaner, un énantiomère purifié d’afoxolaner aux propriétés insecticides et acaricides, est associé à l’éprinomectine et au praziquantel, composés nématodicides et cestodicides, dans NexGard^®^ Combo, une nouvelle formulation endectoparasiticide topique pour chats. L’efficacité de cette formulation a été évaluée contre *Otodectes cynotis* dans deux études de laboratoire menées en Afrique du Sud et aux États-Unis avec des isolats locaux, et dans un essai sur le terrain mené en Europe. Dans chaque étude, les chats ont été répartis au hasard entre un groupe témoin traité par placebo et un groupe traité par la nouvelle formulation. Dans les études de laboratoire, les chats ont été traités à la dose minimale recommandée, et dans l’essai sur le terrain, les chats ont été traités à la dose indiquée sur l’étiquette. Tous les chats inclus ont été diagnostiqués positifs pour *O. cynotis* avant le traitement par otoscopie. La principale variable d’efficacité était une comparaison du nombre d’*O. cynotis* vivants, collectés dans les deux conduits auditifs de tous les chats, dans le groupe traité et dans le groupe témoin, un mois après le traitement. L’efficacité de la nouvelle formulation topique a dépassé 97 % dans les trois études. Ces études ont démontré la grande efficacité de NexGard^®^ Combo chez les chats pour le traitement des infestations par *O. cynotis*. Aucune anomalie de santé n’a été attribuée au traitement dans aucune des études.

## Introduction

The ear mite *Otodectes cynotis* is one of the major ectoparasites of cats and a primary cause of otitis externa [13, 33]. In a multi-centric survey conducted on domestic cats randomly presented in veterinary clinics for reasons unrelated to parasitic disease, in seven countries in Southern and Central Europe, ear mites were diagnosed in 17.4% of 1519 cats [4]. In another survey conducted in Greece, 25.5% of domestic cats were diagnosed ear mite-positive [27]. In this study, mites were found especially in kittens and roaming animals. *Otodectes cynotis* is pathogenic in young cats, dogs and ferrets, and is directly transmitted from one animal to another [19, 23]. It was identified as the primary cause of otitis externa in 53.3% of 187 stray cats [20]. *Otodectes cynotis* can also go unnoticed at an early stage: 14% of kittens up to 6 months in urban areas in Greece were diagnosed ear mite-positive without signs of otitis externa [17]. The correlation between clinical signs and number of *O. cynotis* can be inconsistent: one or two mites have been seen to cause severe otitis lesions with large amounts of dark cerumen, while 50 to 100 mites have been found in clean ear canals [8].

At clinical examination, *O. cynotis* infested ear canals typically reveal erythema and dark brown cerumen. Adult mites, moving white organisms of 0.3–0.5 mm can be visible with the naked eye by otoscopy or on swabs of cerumen and debris from ear canals. Occasionally, the infestation leads to intense irritation and secondary bacterial and/or fungal infection. Otohematoma is also a common consequence of such infestation in cats [8, 20, 22, 27].

Several curative treatments are available, mainly in the form of a macrocyclic lactone topical endectocide [3, 10, 12, 24, 25]. Preventive treatment with a topical formulation containing fipronil, (S)-methoprene, eprinomectin and praziquantel has also been demonstrated [5]. More recently, the isoxazoline class of compounds has brought new curative solutions against mites. Afoxolaner efficacy has been demonstrated against *Sarcoptes, Demodex* and *Otodectes* infestations in dogs [6, 7, 9, 14, 16]. In cats, fluralaner, another isoxazoline compound was demonstrated efficacious against *O. cynotis* infestations in cats alone or in combination with moxidectin [28], as well as sarolaner, alone or in combination with selamectin [1, 26]. Off-label use of oral afoxolaner, a dog-only product (Nexgard^®^), has been found to be efficacious against *O. cynotis* infestations in cats [18].

Esafoxolaner is the purified and active (S)-enantiomer of afoxolaner, a novel compound with insecticidal and acaricidal properties. NexGard^®^ Combo, a novel topical combination of esafoxolaner, eprinomectin and praziquantel was developed for cats with the aim of offering a wide spectrum of antiparasitic activity.

This article describes three studies performed to assess the efficacy in cats of one application of the novel esafoxolaner, eprinomectin and praziquantel formulation for the treatment of *O. cynotis* infestations. Two studies were run under laboratory conditions with induced or natural infestation models, respectively in South Africa and in the United States; one study was run on naturally infested domestic cats in the field, in Hungary and Greece.

## Materials and methods

### Ethics

The study protocols were reviewed and approved by the Sponsor’s and local Institutional Animal Care and Use Committees. Cats were managed and handled with due regard for their wellbeing.

### Study designs

The three studies were conducted in accordance with Good Clinical Practices as described in International Cooperation on Harmonization of Technical Requirements for Registration of Veterinary Medicinal Products (VICH) guideline GL9.

The three studies were conducted under a negative-controlled and randomized design, with cats randomly allocated to two groups, a novel formulation-treated group, and a placebo-treated group.

The *O. cynotis* efficacy assessment was based on comparison of live mites counted in the ear canals of negative control and treated animals one month after treatment.

Personnel involved with evaluation of safety and efficacy, and the owners in the field study, were unaware as to treatment assignments.

The main differences in context and design of the three studies are summarized in Table 1.

**Table 1:** Study contexts and designs.

Studies	Lab #1	Lab #2	Field
Model	Induced infestation	Natural infestation	Natural infestation
Location/date	South Africa/Dec 2015 – March 2016	Michigan, USA/Dec 2018 – Jan 2019	Europe/Oct 2018 – Apr 2019
*O. cynotis* strain origin	South Africa	USA	Hungary and Greece
Variable used for randomization	Bodyweight	Pre-treatment otoscopic live mite count	Order of presentation
Inlusion criteria	Both ear canals positive for mites	Both ear canals positive for mites	At least 1 motile ear mite in 1 ear canal
Number of treated/control cats	8/8	10/10	32/33 sentinels*, 54/61 total*
Treatment dose	Minimum dose of 0.12 mL/kg	Minimum dose of 0.12 mL/kg	Commercial dose
Main variable of efficacy	Live *O. cynotis* counts collected in ear canals, Day 28	Live *O. cynotis* counts collected in ear canals, Day 32	Live *O. cynotis* counts collected in ear canals of sentinel animals, Day 30 (±3)
Secondary efficacy evaluations	Otoscopic assessment (scoring of debris/cerumen and live mites), Days −7, 7, 14, 21, 28	None	Otoscopic assessment (scoring of pruritus and quality/quantity of cerumen), Day 0, Day 30 (±3)

* In the field trial, all cats in a household (total) were treated, one cat per household (sentinel) was evaluated for efficacy and safety, all other cats for safety.

### Ear mite infestation models

In Lab #1, with an induced infestation model, mites were harvested from local donor cats naturally infested with *Otodectes*. Mites were collected by ear flushing and approximately 100 mites were directly transferred into each ear canal of the study cats, under light sedation. Adequate infestation was confirmed by otoscopical assessment when live mites had established in both ear canals. The study was run in three phases as adequate numbers of cats were diagnosed infested. The duration between infestation and treatment was 5, 10 and 16 weeks, respectively.

In Lab #2 and the Field study, with natural infestation models, *Otodectes* naturally infested cats were selected on the basis of positive ear mite diagnosis by otoscopical observation.

### Treatment

Cats were treated once on Day 0. The treatments were applied directly to the skin, after parting the hair, on one spot in the midline of the neck between the base of the skull and the shoulder blades.

In the laboratory studies, cats assigned to the placebo control group were treated with mineral oil at 0.12 mL/kg. Cats assigned to the treated group received a topical application of the novel formulation at the minimum recommended dose of 0.12 mL/kg, delivering 1.44 mg/kg esafoxolaner, 0.48 mg/kg eprinomectin and 10.0 mg/kg praziquantel. Lab #1 was performed at an early stage of product development, using an experimental formulation with identical concentration and composition of active ingredients, but with some differences in the solvent system, in comparison to the final novel formulation which was defined later, and used in Lab #2 and in the Field study.

In the Field study, cats assigned to the treated group received a topical application of NexGard^®^ Combo in the commercial applicator, at the recommended label dose of 0.3 mL/0.8 to <2.5 kg, or 0.9 mL/2.5 to <7.5 kg, delivering 1.44–4.5 mg/kg esafoxolaner, 0.48–1.50 mg/kg eprinomectin and 10.0–31.1 mg/kg praziquantel; cats assigned to the control group were treated with mineral oil at 0.3 mL/0.8 to <2.5 kg, or 0.9 mL/2.5 to <7.5 kg.

### Ear mite counts and ear mite level assessments

In the three studies, a quantitative assessment of ear mites by ear canal mite collection was performed one month after treatment, under sedation. Ear canal content collection was performed by swabbing with cotton buds, and/or by filling with an aqueous solution to melt the ear canal content until clean. The swabbed materials and melted solutions were collected and drained into a sieve. The materials were examined for live mite identification and count, counts included immature and adult forms.

In Lab #1, semi-quantitative ear mite level assessment was also performed weekly via otoscopical examination, as a secondary objective.

### Otoscopical and clinical examinations for otitis signs

Otoscopical and clinical examinations for otitis signs were performed in Lab #1, and the Field study.

The scoring system used for otoscopical and clinical assessments is described in Table 2.

**Table 2:** Otoscopical and clinical scoring.

	Ear mite level	Debris/Cerumen	Pruritus
Score 0	0 mite	Normal, absent	Absent
Score 1	1–4 mites	Clear, low volume	Mild, no skin alteration of external ear
Score 2	5–10 mites	Brown, medium volume	Moderate, mild skin alteration of external ear
Score 3	>10 mites	Dark, large volume	Marked, pronounced skin alteration of external ear
Applied in:	Lab #1	Lab #1, field	Field

In Lab #1, otoscopical examinations were performed before treatment and on Days 7, 14, 21 and 28 (before the ear canal content collection procedure) for ear mite and debris/cerumen level assessments. In Lab #2, no otoscopical examinations were performed for otitis signs. In the field study, otoscopical examinations were performed on Days 0 and 30 (before ear canal collection) for debris/cerumen and pruritus level assessment.

### Statistical analysis

In the three studies, to compute percent *O. cynotis* efficacy, the geometric means of the total of adult and immature live mite counts in both ear canals were calculated by group. The geometric means were computed by taking the anti-logarithm of the average of the log (mite count + 1) and then subtracting 1. Percent effectiveness with respect to the control group was calculated using the formula ([*C*−*T*]/*C*) × 100, where *C* = geometric mean for the control group, and *T* = geometric mean for the treated group.

The log-counts of the treated group were compared to the log-counts of the control group using an *F*-test adjusted for the allocation blocks used to randomize the animals to the treatment groups. The MIXED procedure in SAS Version 9.4 was used for the analysis, with treatment group listed as a fixed effect and the allocation blocks listed as a random effect. All testing was two-sided at *α* = 0.05 significance level. Lab #1 was conducted using three phases. The effect of the phases was evaluated by modifying the above analysis of the logarithm of the total mite counts + 1 in the Mixed procedure by adding phase and treatment-by-phase interaction as fixed effects.

## Results

### Ear mite counts

The results of ear mite counts following ear canal collections, one month after treatment, are summarized in Table 3.

**Table 3:** Ear mite counts following ear canal flushing in Lab #1, Lab #2 and the Field study, one month after treatment.

	Control group		Treated group		% Efficacy	*p*-value
	*n*	Geo mean (range)	*n*	Geo mean (range)		
Lab #1	8	226.6 (16–2225)	8	6.4 (0–54)	97.2	<0.001
Lab #2	10	65.1 (4–630)	10	0.1 (0–1)	99.9	<0.0001
Field Study	33	139.6 (2–1548)	32	3.7 (0–136)	97.4	<0.0001

Lab #1: the live ear mite counts on Day 28 revealed adequate infestation of the untreated control cats, and a high level of efficacy in the treated group with 97.2% reduction (0, 0, 1, 2, 26, 27, 34, 54 live mites were found in the eight cats of the treated group).

Lab #2: the live ear mite counts on Day 32 revealed adequate infestation of the untreated control cats and a high level of efficacy in the treated group with 99.9% reduction (only one live mite was found in the ten cats of the treated group).

Field trial: The live ear mite counts on Day 30 revealed adequate infestation of the untreated control cats and a high level of efficacy in the treated group with 97.4% reduction (0–136 live mites were found in the 33 cats of the treated group).

### Ear canal otoscopical examinations

None of these observations were supported by a statistical analysis and should be considered purely descriptive.

**Figure 1:**
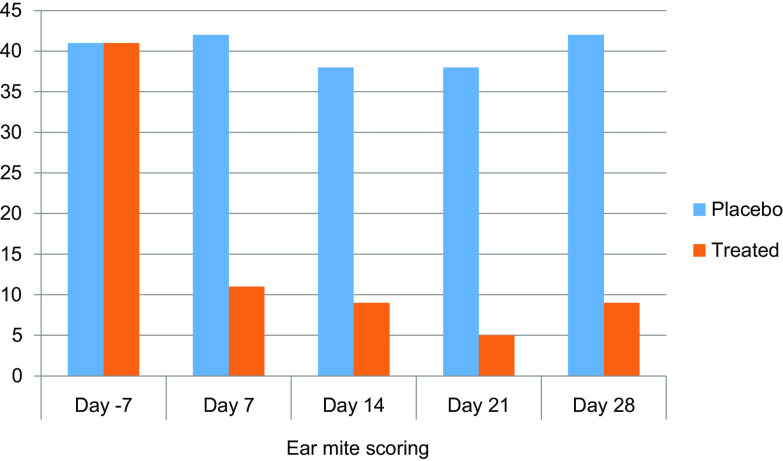
Ear mite otoscopical scoring in Lab #1. Sum of individual otoscopical scores of live mites inclusive of both ears of each cat per group (*n* = 8).

**Figure 2:**
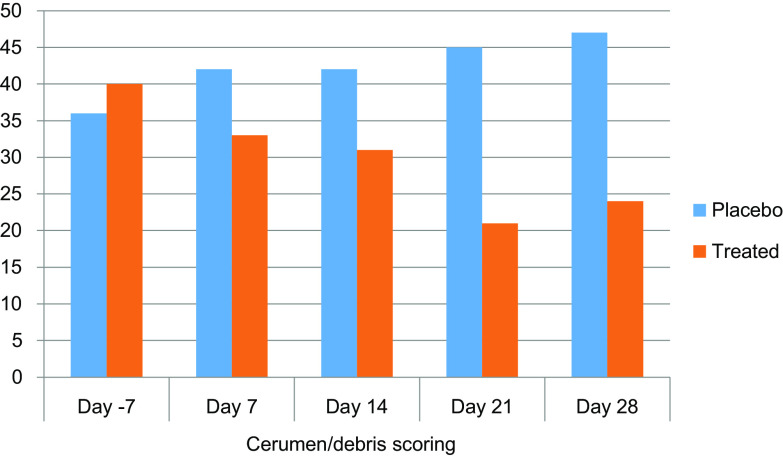
Debris/cerumen scoring in Lab #1. Sum of individual otoscopical scores of debris/cerumen inclusive of both ears of each cat per group (*n* = 8).

In Lab #1, the results of otoscopical assessments are illustrated in Figures 1 and 2. The treatment effect was visible on the otoscopical semi-quantitative mite level assessment performed weekly for 1 month (Fig. 1). Overall, it remained unchanged in the placebo group; in the treated group, it had dropped to approximately a quarter of the baseline level on the first week after treatment and remained steady until the end of the month. The otoscopical semi-quantitative mite assessments in the treated group in comparison to mite counts following ear canal flushing (Table 3), show that quantification of ear mites by otoscopy is relevant, however less accurate, especially by its limitation to reflect high numbers of ear mites (no statistical analyses were performed to compare the two methods).

The treatment effect was visible on the debris/cerumen scoring (Fig. 2). Over the month of the study, the overall level of cerumen/debris had slightly increased in the placebo group, while it had approximately halved in the novel formulation-treated group.

In the Field study, the course of otitis signs between Day 0 and Day 30 is described in Table 4. One month after treatment, in the novel formulation-treated group, the debris/cerumen level had improved in a majority of cats (28 of 32) and had cleared in a third of the cats (10 of 32); the pruritus level had improved in a majority of cats (27 of 32), and had cleared in half the cats (17 of 32). In the untreated control group, the otitis signs remained the same in about two third of the cats, other cats improved, and a smaller number worsened.

**Table 4:** Course of otitis signs in the Field study, one month after treatment

	Debris/Cerumen				Pruritus			
	Worse	Same	Improved	Cleared	Worse	Same	Improved	Cleared
Control group (*n* = 33)	3 (2)*	24	6 (0)	0 (0)	3 (2)*	20	10 (3)*	2 (0)
Treated group (*n* = 32)	0 (0)	4	28 (4)*	10 (1)*	1 (0)	4	27 (3)*	17 (0)

Worse: otoscopical scoring was higher on Day 30 than on Day 0.

Same: otoscopical scoring for both ears was identical on Days 0 and 30.

Improved: otoscopical scoring was lower on Day 30 than on Day 0.

Cleared: otoscopical scoring was 0.

* In brackets = number of cats for which only one ear improved, worsened or cleared.

## Discussion and conclusions

The results of these three studies provide a robust demonstration of the high level of efficacy of NexGard^®^ Combo for the treatment of *O. cynotis* infestations, in induced and natural infestation models in laboratory and field studies, in Europe, the United States, and South Africa, with efficacy results of a single treatment exceeding 97% each time.

It was observed and confirmed in the untreated cats that the level of infestation in ear mite infested cats is highly variable and can range from a few acarians to several hundred per ear canal, without a clear or consistent intensity correlation with otitis signs [8], in natural or induced laboratory models, and in the field.

In Lab #1 and the Field study, the studies with highest infestation levels, even though treatment efficacy was demonstrated, some of the treated cats still had a significant number of mites in their ear canals one month after treatment. The lifecycle of *O. cynotis* is 18–28 days [33], meaning that all development forms of the parasite had been exposed to the acaricide compound in the treated groups before mite collection. It is, nevertheless, unclear whether the full *O. cynotis* development cycle was broken for most infested cats of the group, and whether the observed live mites had survived the acaricide treatment over the month or were new mites from unkilled eggs hatching near the end of the month, and whether these live mites would have survived and started a new cycle or died later. The otitis signs observed in these two studies confirm that the novel formulation treatment provided a clear improvement of clinical signs; however, their level of improvement was not as high as the measured elimination of mites. In the absence of studies verifying the effect of a single treatment with the novel formulation over a period exceeding one month, it might be advisable to administer a second treatment after one month for highly infested cats, or for cats for which otitis signs had not fully cleared one month after treatment.

This novel association of esafoxolaner, eprinomectin and praziquantel offers a broad spectrum of efficacy against the main parasites of cats including ecto- and endoparasites [15, 21, 29–31]. The control of multiple and various concurrent parasitic infestations by a range of cat parasites is important for cats but also for public health [2, 11, 32]. Co-infestation of ecto-parasites and endoparasites was demonstrated in 14% of 1519 cats and *O. cynotis* was the most prevalent ectoparasite (17.4%) of cats [4].

In addition to a high level of efficacy and safety, owner and cat compliance is an important feature of success for this type of therapeutic approach, and the simple conditions of use and of treatment application of this product should guarantee a high level of compliance.

## Competing interest

The work reported herein was funded by Boehringer-Ingelheim. The authors are current employees of Boehringer-Ingelheim Animal Health or external organizations. Other than that, the authors declare no conflict of interest. This document is provided for scientific purposes only. Any reference to a brand or trademark herein is for information purposes only and is not intended for any commercial purposes or to dilute the rights of the respective owners of the brand(s) or trademark(s). NexGard^®^ is a registered trademark of the Boehringer Ingelheim Group.
